# Supplementary material for: Structural compatibility enables functional co-assembly of Kir2.1 channels and the voltage-sensing domain of voltage-sensitive phosphatases
Source: Pflugers Arch. 2026 Nov 11;478(12):97. doi: 10.1007/s00424-026-03227-3 (PMC13755035; doi:10.1007/s00424-026-03227-3)
Supplement: Supplementary file 1 [file 424_2026_3227_MOESM1_ESM.pdf]

Structural compatibility enables functional co-assembly of Kir2.1 channels and the voltage-sensing domain of voltage-sensitive phosphatases

Supplementary Information

Anagha Gopalakrishnan Nair[1#], Philipp Rühl[1#], Lina Seeber[1], Toshinori Hoshi[2], Roland Schönherr[1], Stefan H. Heinemann[1]*

# Shared first authorship.

1 Center for Molecular Biomedicine, Department of Biophysics, Friedrich Schiller University Jena and Jena University Hospital, Jena, Germany.

2 Department of Physiology, University of Pennsylvania, Philadelphia, USA.

*Corresponding author:

Center for Molecular Biomedicine, Department of Biophysics,
Friedrich Schiller University Jena & Jena University Hospital,
Hans-Knöll-Str. 2, 07745 Jena, Germany.

stefan.h.heinemann@uni-jena.de ORCID: 0000-0002-4144-0251

anagha.gopalakrishnan.nair@uni-jena.de ORCID: 0009-0002-4857-6554

philipp.ruehl@uni-jena.de ORCID: 0000-0002-3554-3107

lina.seeber@uni-jena.de

hoshi@pennmedicine.upenn.edu ORCID: 0000-0002-0702-1918

roland.schoenherr@med.uni-jena.de ORCID: 0000-0003-0633-0775

Supplementary Figures

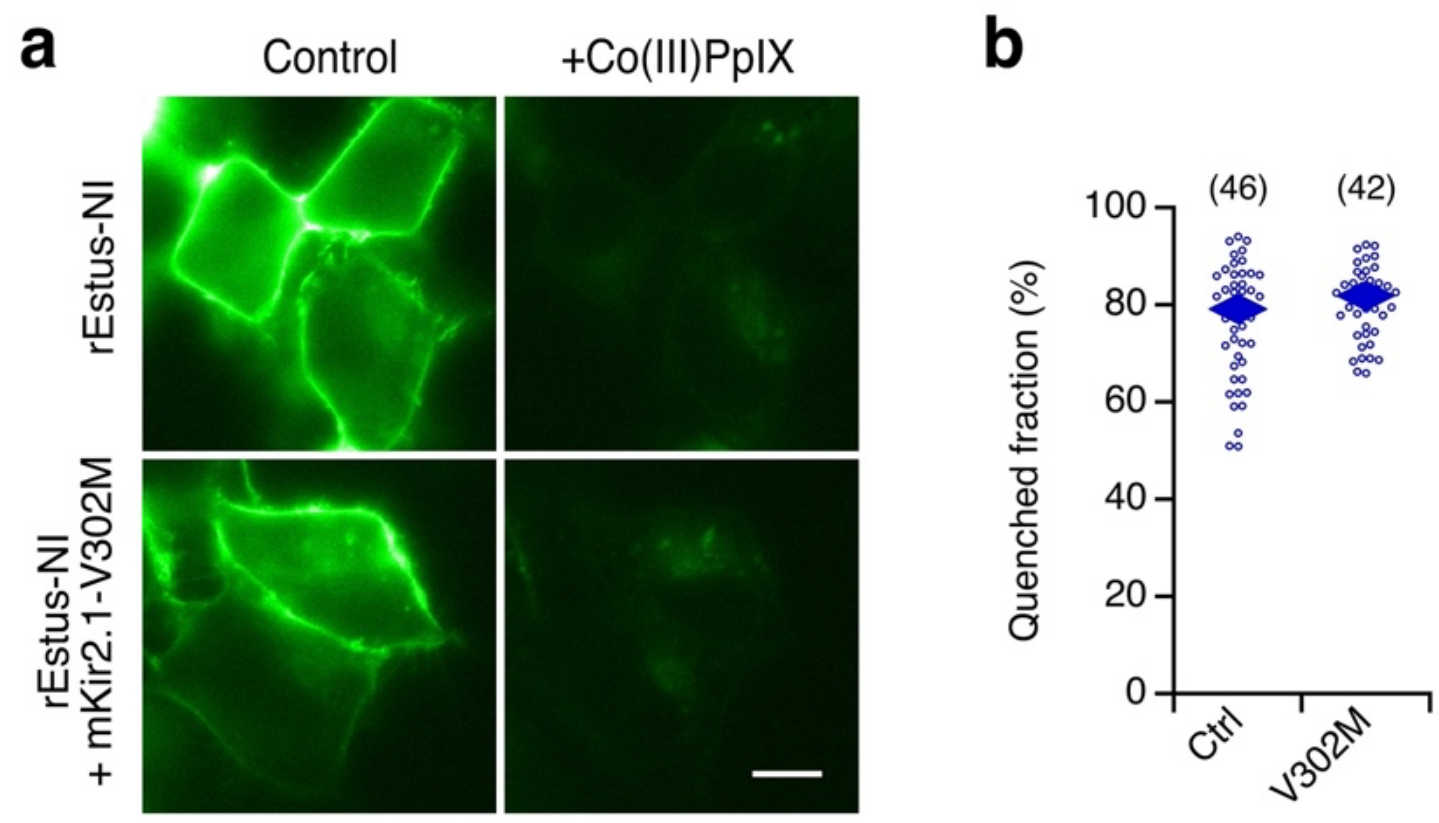

Supplementary Figure 1. rEstus-NI trafficking is not altered by coexpression of mKir2.1-V302M. a Fluorescence images of HEK293T cells expressing either rEstus-NI alone or rEstus-NI together with mKir2.1-V302M under control conditions (*left*) and after application of 10 µM Co(III)PpIX (*right*). Co(III)PpIX quenches the fluorescence of extracellularly accessible rEstus-NI [1]. **b** Quantitative analysis of images as in (a) by plotting the percentage of remaining fluorescence after Co(III)PpIX application. Rhombs indicate the medians of the distributions. The membrane targeting of rEstus-NI was similar in the absence (77.7 ± 1.7%) and presence mKir2.1-V302M (80.4 ± 1.1%; p = 0.09, t test).

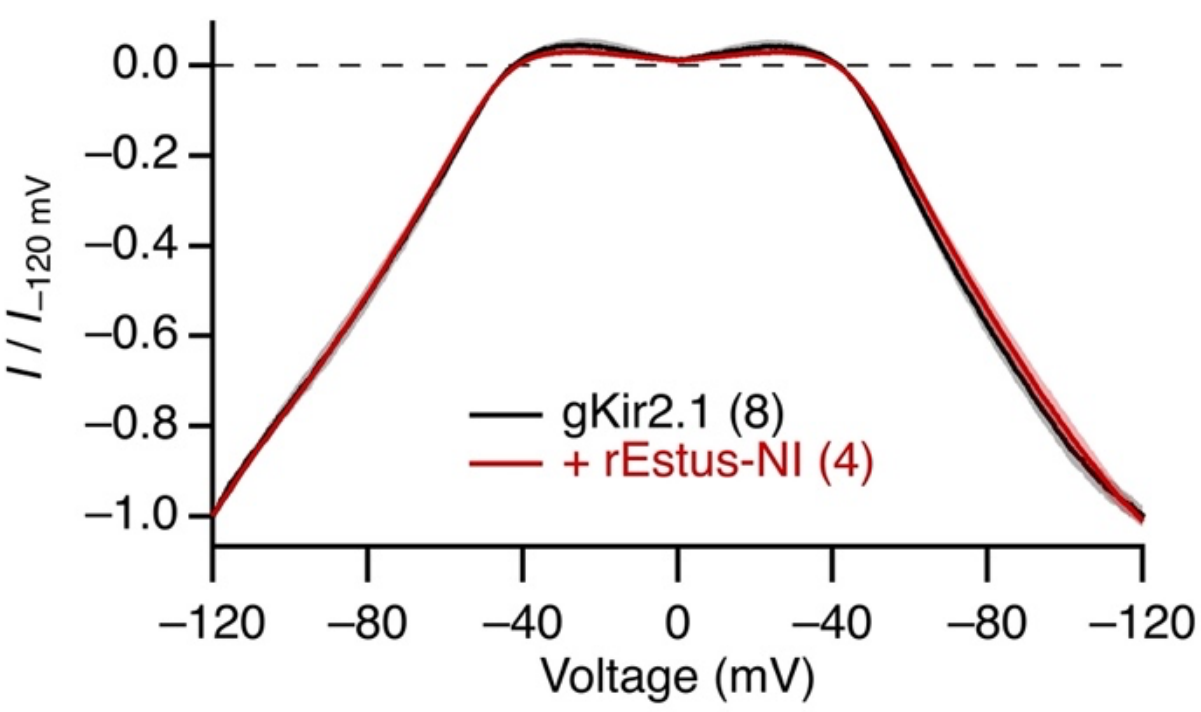

Supplementary Figure 2. Co-expression of rEstus-NI has no obvious impact on currents mediated by gKir2.1. Superposition of mean current responses to bipolar voltage ramps (–120 to 0 mM and return to –120 mV in 1 s) obtained from HEK293T cells expressing gKir2.1 alone (black) or co-expressed with rEstus-NI (red). Currents were normalized to the starting current at -120 mV. Data are means ± SEM in shading, n in parentheses. The reversal potential was -40 mV to make the voltage range -120 to -40 mV, i.e., the range where rEstus-NI undergoes the conformational changes, experimentally accessible. Extracellular solution (in mM): 118 NaCl, 32 KCl, 2 CaCl2, 2 $MgCl_2$, 10 HEPES, pH 7.4 (with NaOH); intracellular solution (in mM): 150 KCl, 10 EGTA, 10 HEPES, pH 7.4 (with KOH).

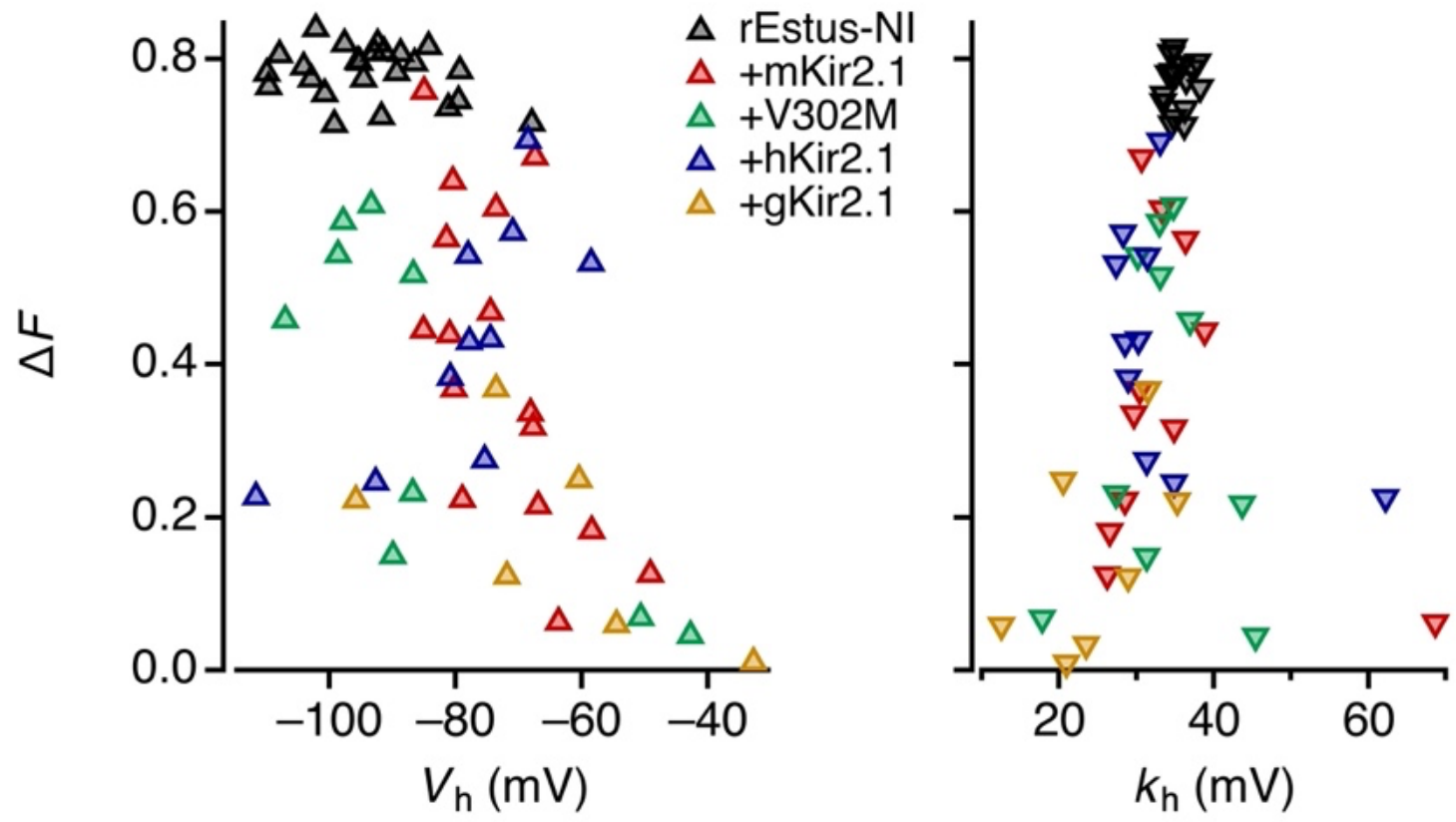

Supplementary Figure 3. Correlation of parameters describing the voltage dependence with the maximal rEstus-NI fluorescence change. Scatter plots of the data shown in Fig. 2 for rEstus-NI alone (black) or co-expressed with one of the indicated Kir2.1 variants (colored). The half-maximal fluorescence change voltage (V_h) tended to move toward more depolarized V_h values as ΔF decreased (*left*), while the slope factor k_h tended to decrease with smaller ΔF values with some outliers (*right*).

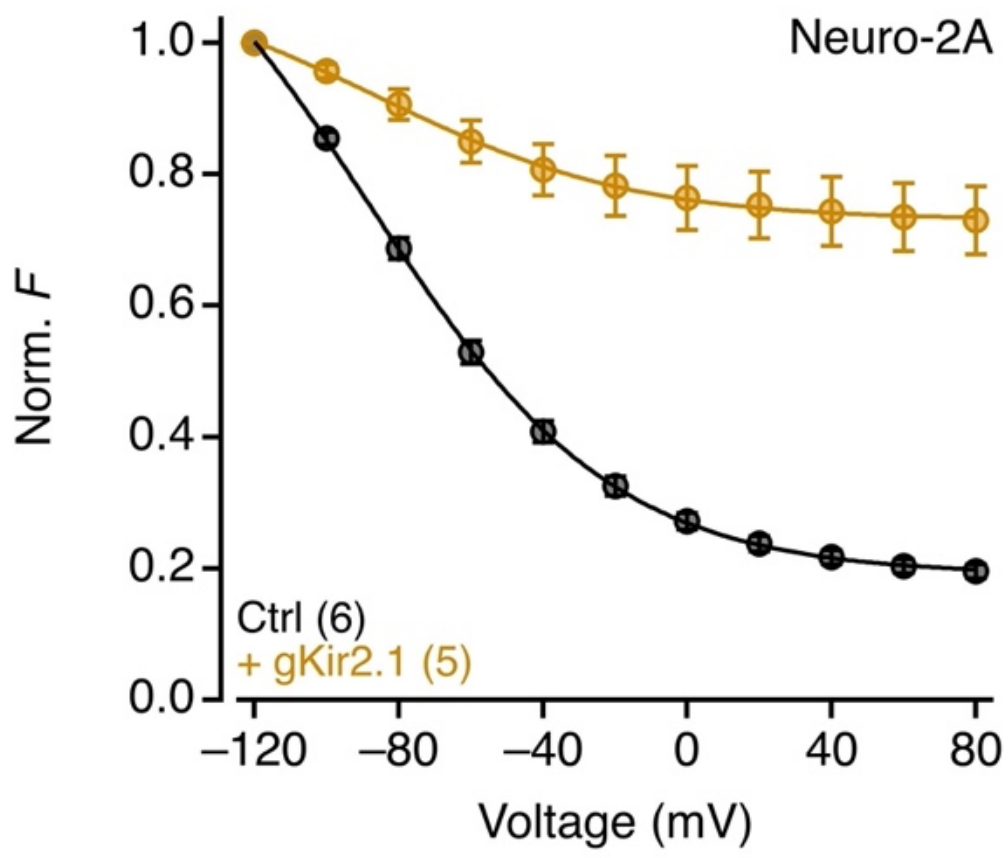

Supplementary Figure 4. Coexpression of rEstus-NI and gKir2.1 in Neuro-2A cells. Fluorescence–voltage (F–V) relationships of rEstus-NI recorded under whole-cell voltage clamp in Neuro-2A cells, normalized to the fluorescence at -120 mV. Data are from rEstus-NI alone (black) and co-expressed with gKir2.1 (orange). Solid curves indicate Boltzmann fits (Eq. 1). Data are means ± SEM; the numbers of cells (n) are indicated in parentheses. ΔF values are 0.83 ± 0.01 for Ctrl and 0.27 ± 0.05 for the coexpression of gKir2.1 (p = 2 10^{-6}). V_h: -88.5 ± 3.4 mV vs. -79.7 ± 4.8 mV; k_h: 33.9 ± 0.7 mV vs. 34.3 ± 7.5 mV (p = 0.16 and 0.95, respectively).

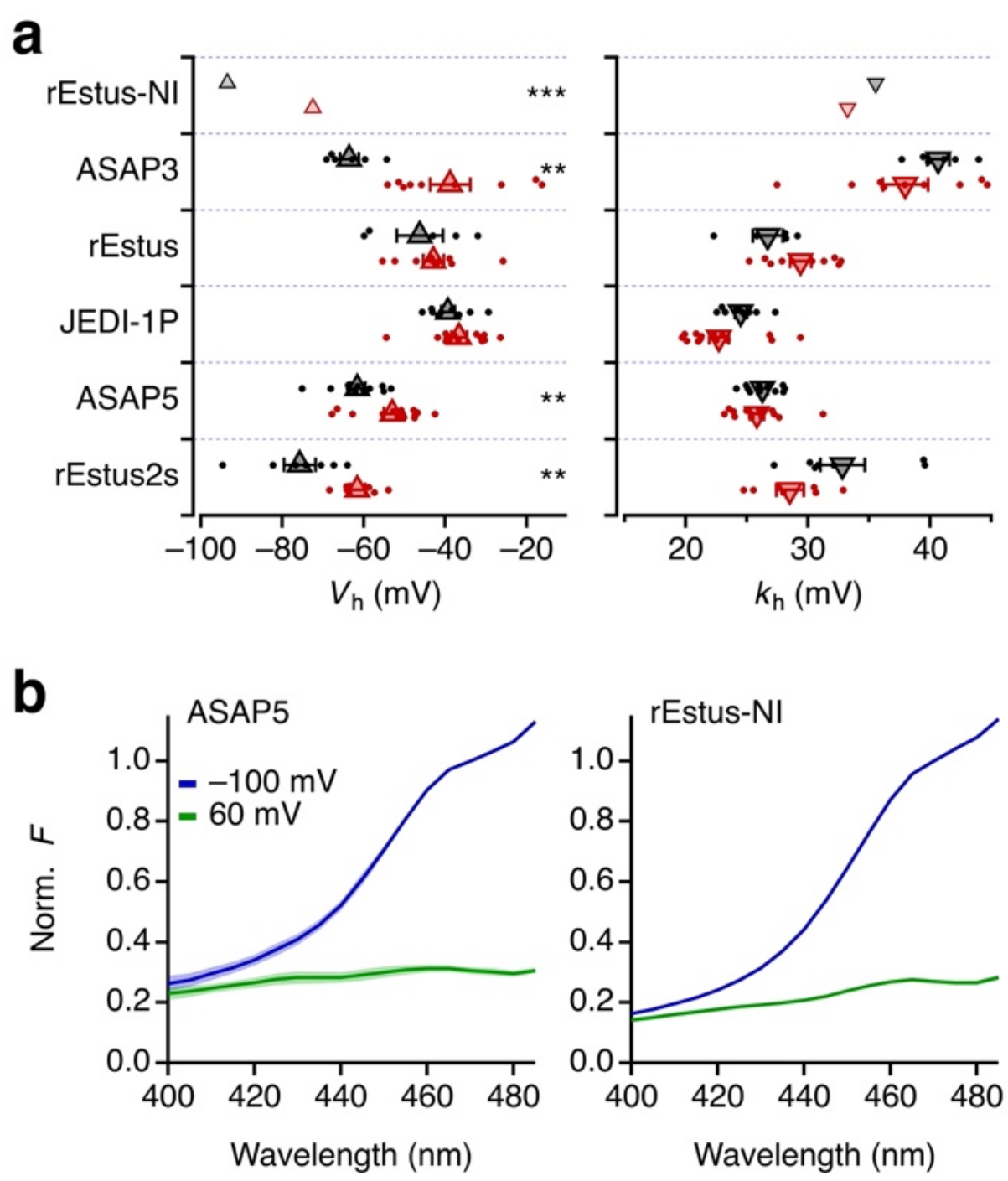

Supplementary Figure 5. Functional GEVI properties. a Voltage of half-maximal GEVI activation (V_h, *left*) and the corresponding slope factors (k_h, *right*) from the data fits shown in Fig. 3a; mean values for rEstus-NI are shown as reference. **b** Excitation spectra, normalized to 470 nm, of ASAP5 and rEstus-NI in HEK293T cells at -100 and 60 mV. Data are means ± SEM in shading, n = 5. The spectra are corrected for wavelength-dependent excitation light intensity variations.

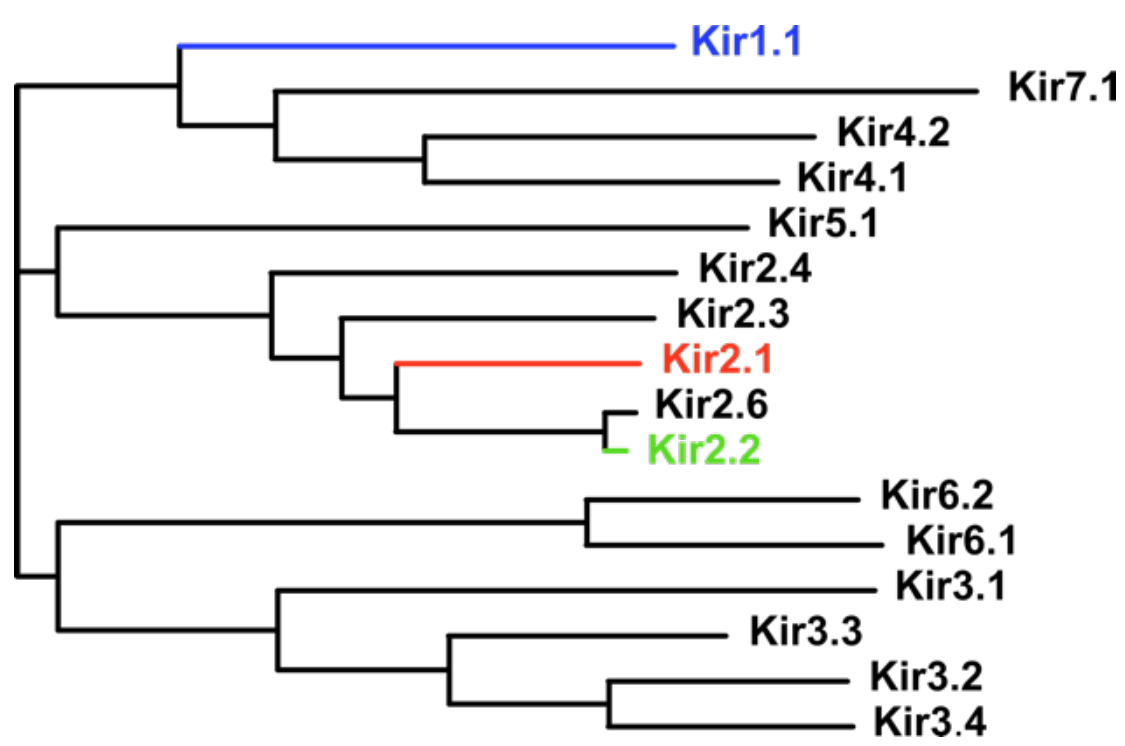

Supplementary Figure 6. Phylogenetic tree of inward rectifier channel subunits. Protein sequences of human inward rectifier channel subunits were retrieved from UniProt and aligned using the UniProt multiple sequence alignment tool. The resulting phylogenetic tree was visualized using iTOL (Interactive Tree of Life: itol.embl.de). The colored variants were examined in this study. Alignments of the TM1 and TM2 transmembrane regions revealed an identity between human Kir2.1 and Kir2.2 of 53.6 and 67.9%, respectively, while these scores were 40 and 54.4% for Kir2.1 compared with Kir1.1.

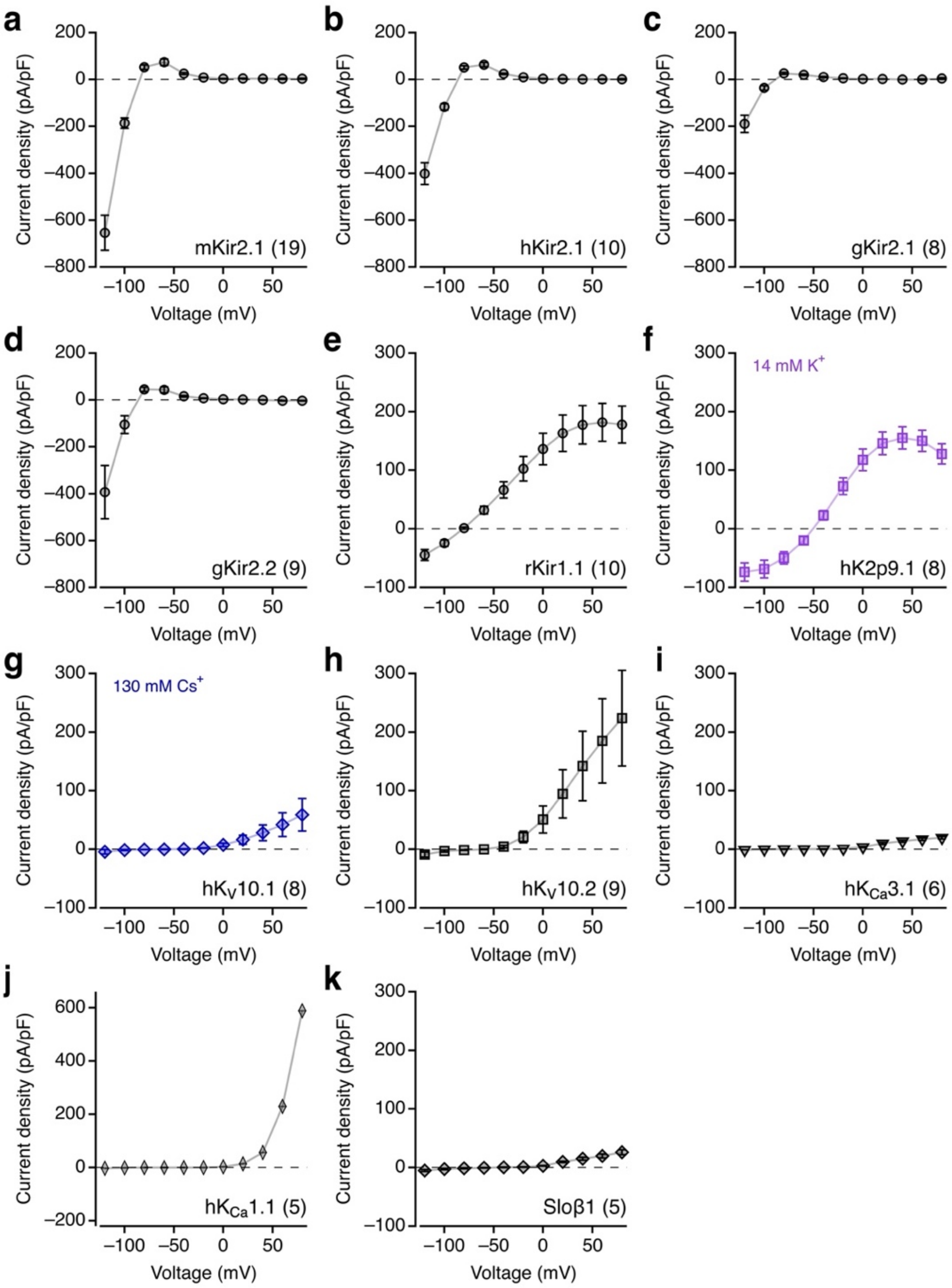

Supplementary Figure 7. Current recordings from HEK293T cells expressing rEstus-NI along with various K^+ channel types. a-k Mean current densities (mean current during the second half of the depolarization segment, see Fig. 2b, divided by the cell capacitance) as a function of voltage from HEK293T cells expressing rEstus-NI along with the indicated K^+ channel types. Data are means ± SEM, *n* in parentheses. Straight lines connect the data points. Because very large currents distort the real applied voltage due to limited series resistance compensation, currents for hK2p9.1 (**f**) and $hK_V10.1$ (**g**) were measured with pipette solutions containing 14 mM K^+ (**f**) or 130 mM Cs^+ (**g**) instead of 140 mM K^+ ions. Note that the intracellular solutions were buffered with 10 mM EGTA, thus eliminating most of the free Ca^{2+} ions. Therefore, $hK_{Ca}3.1$ (**i**) does not show current above the leak level and $hK_{Ca}1.1$ (**j**) only starts activating above about 40 mV. In the presence of the BK_{Ca} β1 subunit (**k**), only current endogenous to HEK293T cells is observed.

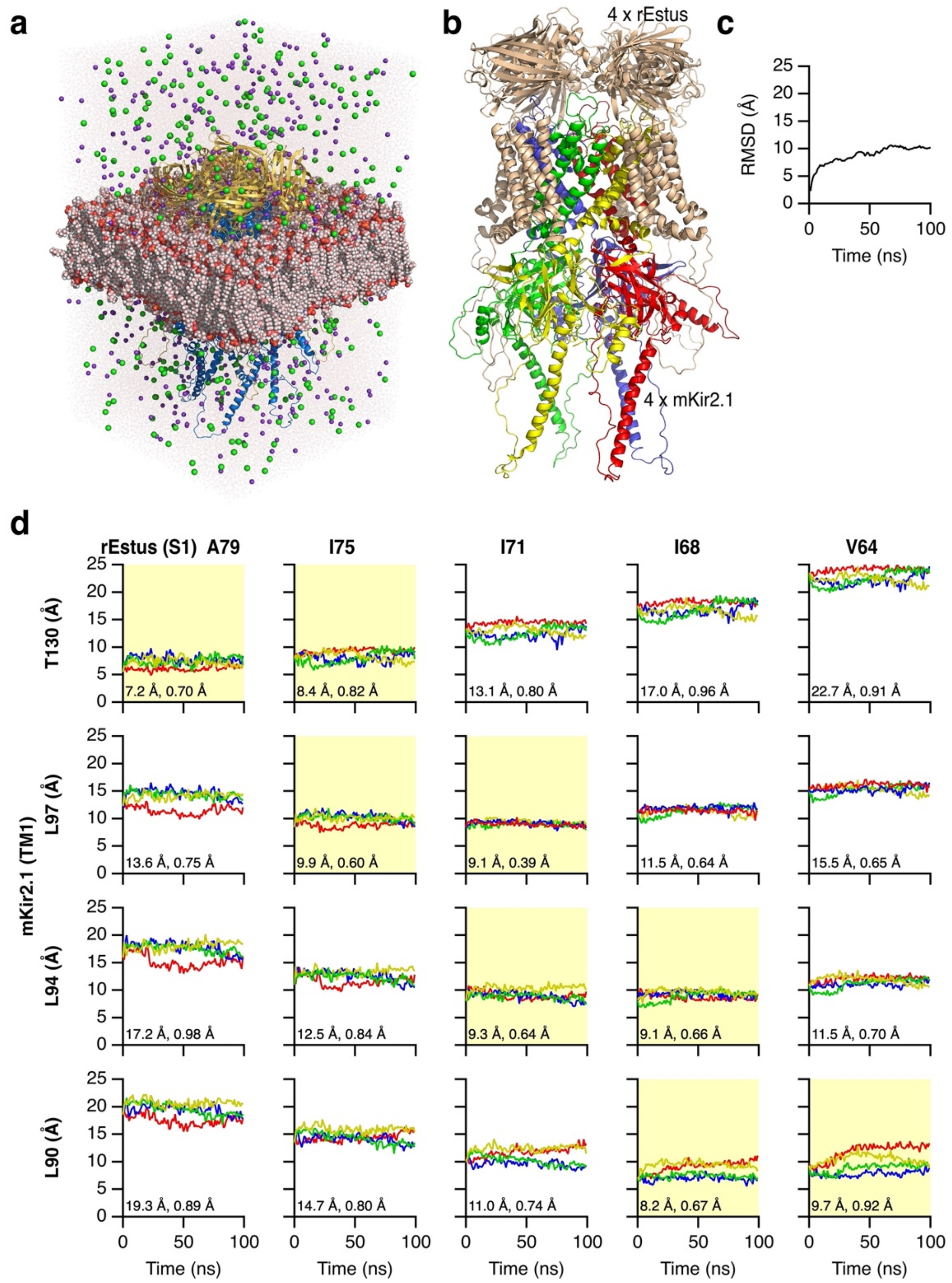

Supplementary Figure 8. Molecular dynamics simulation of an octameric rEstus–mKir2.1 assembly in a lipid membrane. a Simulation box consisting of four rEstus polypeptides, four mKir2.1 polypeptides, a lipid membrane, water, and 150 mM KCl. **b** Side view of the octameric rEstus–mKir2.1 complex. rEstus is shown in gold, the four units of mKir2.1 are colored. **c** Root mean squared deviation (RMSD) of all protein atoms during the simulation of 100 ns indicates that the complex reaches an equilibrium structure after about 60 ns. **d** Distances between the alpha carbon (CA) atoms of the indicated residues in mKir2.1 and the voltage-sensor domain of rEstus as a function of simulation time. Results from the four subunits are indicated in colors matching the color-coding of (**b**). The numbers in the panels indicate the mean distance in all four subunits and the average SEM of the simulation time courses. Yellow shading indicates

residue pairs with the two lowest mean distances. Lowest SEM values indicate a firm interaction between the respective amino acid residues: T130-A79, L97-I71, L94-I71, L90-I68.

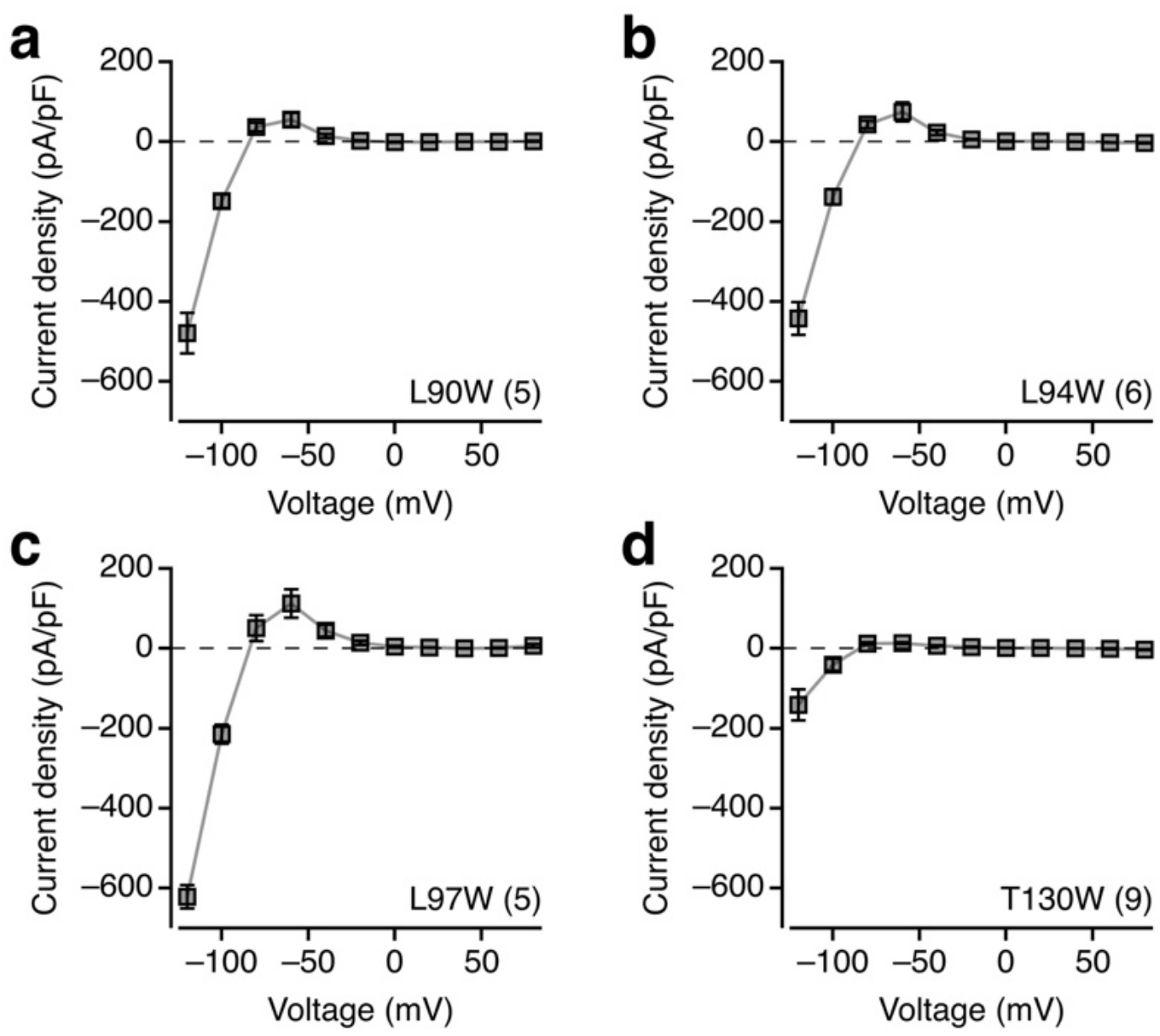

Supplementary Figure 9. Current recordings from HEK293T cells expressing rEstus-NI along with mKir2.1 mutants. a-d Average current densities (mean current during the second half of the depolarization segment, see Fig. 2b, divided by the cell capacitance) as a function of voltage from HEK293T cells expressing rEstus-NI along with the indicated mutants of mKir2.1. Data are means ± SEM, *n* in parentheses. Straight lines connect the data points.

References

1. Muthukumar, A.S., et al., *Cobalt protoporphyrin IX is a potent quencher of fluorescent proteins.* Under review, 2026.
